# Isolated fallopian tube torsion with paraovarian cysts: a case report and literature review

**DOI:** 10.1186/s12905-021-01483-2

**Published:** 2021-09-28

**Authors:** Liang Qian, Xue Wang, Dingheng Li, Songyi Li, Jiashan Ding

**Affiliations:** 1 Department of Gynecology, Hangzhou Women’s Hospital, Hangzhou, 310008 Zhejiang China; 2 Nanjing Medical University, Nanjing, 211100 Jiangsu China; 3 Department of Obstetrics and Gynecology, The First Affiliated Hospital of Nanchang University, Nanchang, 330006 China

**Keywords:** Case report, Isolated fallopian tube torsion, Paraovarian cyst, Salpingectomy, Ultrasound

## Abstract

**Background:**

Isolated fallopian tube torsion (IFTT) is a rare cause of gynecological acute abdomen, is easily misdiagnosed and often has a delay in diagnosis. IFTT with paraovarian cysts is most frequently reported in studies. Here, we reported a patient diagnosed with IFTT associated with a paraovarian cyst, and we conducted a literature review for IFTT, aiming to identify valuable information that will be helpful for diagnosis and treatment for fallopian tube torsions.

**Case presentation:**

A 13-year-old girl presented with a 10-day history of right lower abdominal pain that worsened 2 days before presentation. On presentation, ultrasound showed a 5.8 * 5.5 cm hypoechoic cyst adjacent to the right ovary, and between the cyst and ovary, a tortuous thickened tube was visualized. Laparoscopy revealed a triple torsion of the right fallopian tube with a 6-cm paraovarian cyst, and tubal conservation surgery was performed. The postoperative course was uneventful. Histopathological diagnosis revealed serous papillary cystadenoma.

**Conclusion:**

Paraovarian cystic dilatation often occurs in adolescence and can induce fallopian torsion when the size of the cyst reaches 5-cm. In our review, the median age of patients diagnosed with IFTT with paraovarian cysts was 15 years old, and the main clinical manifestation was emergency abdominal pain. The associated symptoms were variable, and vomiting was the most commonly associated symptom. Salpingectomy was the most common procedure performed; however, timely surgical intervention can effectively avoid salpingectomy.

## Background

IFTT is the torsion of the fallopian tube on its own axis. It is a rare cause of gynecological acute abdomen and was first reported by Bland-Sutton [1]. The estimated prevalence is 1 in 1.5 million women [2]. IFTT is easy to misdiagnosis, and treatment can be delayed due to its rarity of occurrence and nonspecific clinical manifestation. The etiologies for IFTT include intrinsic and extrinsic factors such as anatomic abnormalities, sudden changes in body position, hyperlaxity of the tube and ligament due to gestation or acyeterion drugs, adhesions due to pelvic inflammation and so on [3]. Anatomic abnormalities, including hydrosalpinx, hematosalpinx, paraovarian cyst, tubal tumor, long tubal, and paraovarian cyst, were most frequently reported in previous studies. However, to the best of our knowledge, no systematic review of IFTT with paraovarian cysts currently exists. Herein, in this study, we reported a case of a 13-year-old girl diagnosed with IFTT with a paraovarian cyst and conducted a literature review on this patient with IFTT. The purpose of this study was to determine valuable information that could be helpful for the diagnosis and treatment of fallopian tube torsions.

## Case presentation

**Fig. 1:**
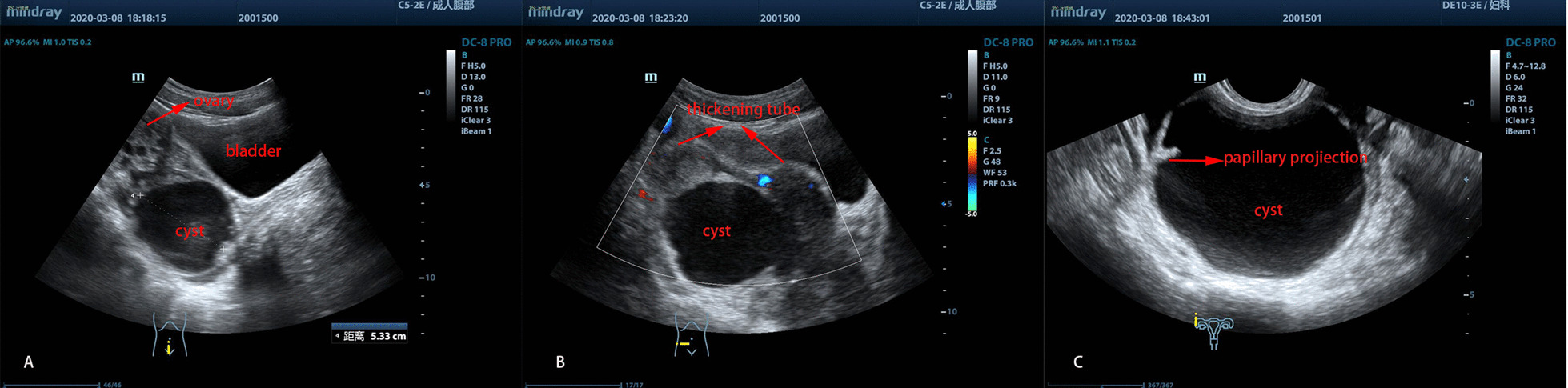
Ultrasonographic identification of a cystic mass in the right lower abdomen **A** a normal ovary with a cystic mass underneath it. **B** Thickening tube beside the cyst. **C** The papillary projection in the cyst wall

A 13-year-old sexually inactive girl was admitted to our department due to a 10-day history of right lower abdominal pain, which worsened the last 2 days before presentation. It was suspected that she had an ovarian torsion and was treated by a local hospital with medication that did not relieve the symptoms. The physical examination suggested lower abdominal tenderness without defense. Pelvic examination revealed normal external genitalia. A cystic mass approximately 6-cm in diameter was palpable behind the uterus. Laboratory evaluations showed normal blood tests. Transabdominal and transrectal ultrasounds showed normal ovaries and a 5.8 * 5.5 cm hypoechoic cyst adjacent to the right ovary, and between the cyst and ovary, a tortuous thickened tube was visualized (Fig. 1A, B). Furthermore, a two-mm papillary projection was also seen on the inner wall of the cyst (Fig. 1C).

**Fig. 2:**
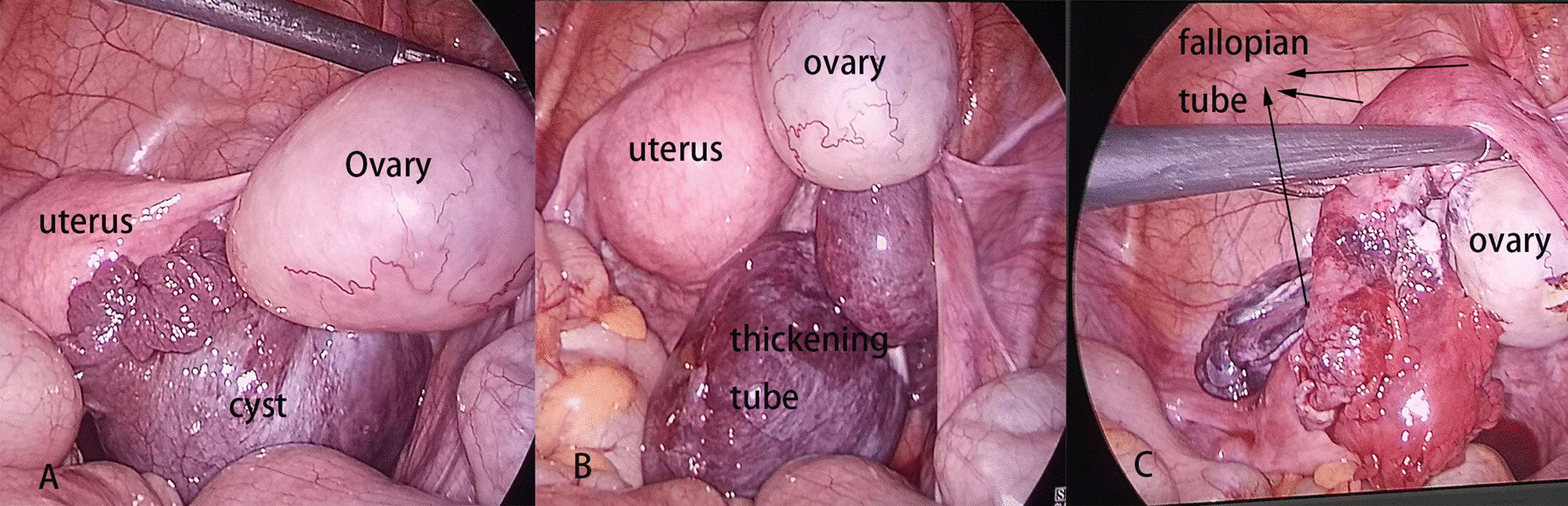
**A** The normal ovary and uterus, with a cystic mass in the middle. **B** The thickening of the torsed tube. **C** The fallopian returned to a pink color after the detorsion of the tube and denucleating the cyst

**Fig. 3:**
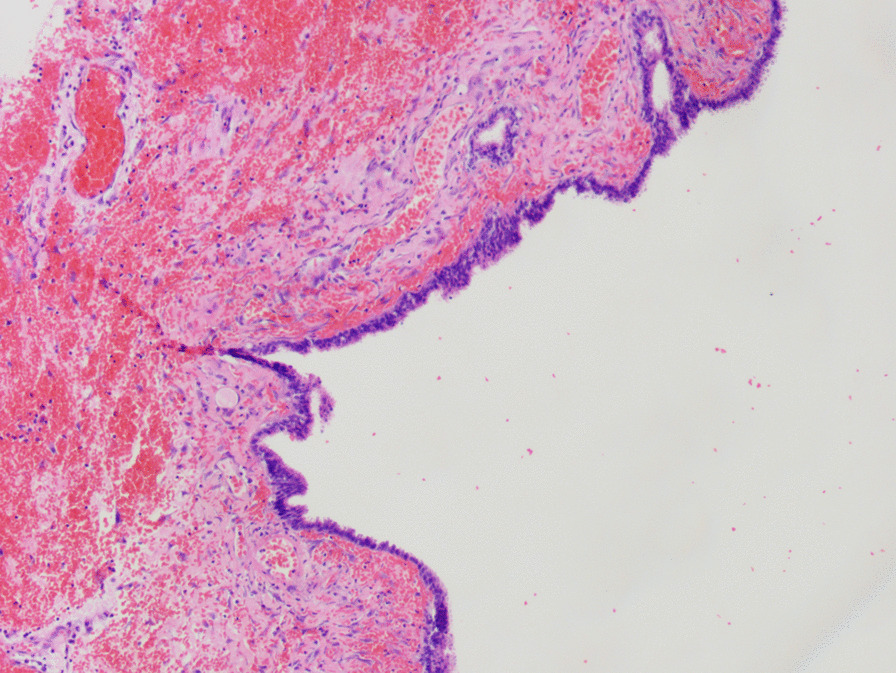
Photomicrograph of the cyst shows serous papillary cystadenoma, accompanied by hemorrhagic infarct on the wall. Magnification × 100

A preliminary diagnosis of IFTT was made, and laparoscopy was performed immediately, which showed a triple torsion of the right fallopian tube associated with a six-cm paraovarian cyst. The fallopian tube appeared dilated and edematous. After the detorsion of the fallopian tube and denucleating the cyst, the right fallopian tube return to a pink color after reperfusion (Fig. 2). The postoperative course was uneventful, and she was discharged 5 days after the operation. The histopathological diagnosis was serous papillary cystadenoma (Fig. 3).

We searched the PubMed database by using the terms “paratubal cyst”, “paraovarian cyst”, “tubal torsion”, “isolated fallopian tube torison”, and “isolated tubal torsion”. After screening all of the publications, cases of IFTT associated with paraovarian cysts written in English were included in the analysis. References without a full-text article were excluded. The details of the patients’ diagnosis and treatment processes, such as age, presentation, degree of pain, blood tests, preoperative diagnosis, surgical approach, and torsion time, were collected and analyzed.

## Results

After the literature search on PubMed, this study included 19 publications regarding IFTT with paraovarian cysts. Among these studies, 17 were case reports (Table 1), and another two were case series without details of the patients [4, 5]. We analyzed 20 patients included for our study. The median age of patients was 15 years old. Four patients had a torsion of the left fallopian tube, while 14 patients had a torsion of the right fallopian tube. Another two patients had bilateral torsions. The mean number of torsion rotations was 2.38 rotations, ranging from one to four rotations. All patients reported abdominal pain, 17 cases had the mode of pain reported, and all patients presented with emergency abdominal pain. The median pain duration time was 3 days (the longest 5 months, the least 4 h). Nine patients presented with accompanying symptoms of vomiting. Micturition abnormalities (n = 1), dyspareunia (n = 1), tachycardia (n = 1), and diarrhea (n = 1) were also present. Except for two previous cases from 1967 and 1972, ultrasonography was performed as the first diagnostic examination in all other cases.

**Table 1:** Details of diagnostic and operative procedures in patients with IFTT + paraovarian cyst

Authors	Case no	Age (years)	Duration of abdominal pain	Mode of pain	Another presentation	Blood test	Image examination	Preoperative diagnosis	Approach	Side	Twist time	Procedure	Measure of cyst	Pathology	Else
Iffy et al. [19]	1	23	2 episode pain during 3 weeks	Severe	–	WBC 8.700/ml	–	Ovarian cyst torsion	Laparotomy	Right	3	Salpingectomy	7.0 × 6.5 × 6.0 cm^3^	Cyst wall contain muscle fibres and layer of low columnar cells	In the puerperium
Barkla [20]	1	22	3-days	Severe	Vomiting + micturition 2 weeks	WBC 12,200/ml	–	Acute-on-chronic salpingitis	Laparotomy	Right	3	Salpingectomy	5 cm in diameter	Tube is distended and infarcted	
Howard et al. [21]	2	23	4 h	Acute	–	Normal	US	Ovarian torsion	Laparotomy	Left	3	Untwisted	5.5 × 4.3 × 3.6 cm^3^	Benign, cuboidal epithelium	At 21 weeks' gestation
Yalcin et al. [22]	2	31	4-days	Intensity during last 2 days	–	Normal	US	Torsion of an ovarian cyst	Laparotomy	Right	2	Salpingectomy	8.2 × 7.4 × 5.7 cm^3^	Cyst wall epithelium	At 34 weeks' gestation
Phupong et al. [23]	1	34	2-days	–	Vomiting	WBC 15,000/ml	US	Twisted left ovarian cyst	Laparotomy	Left	3	Salpingectomy	6 cm in diameter	Paratubal cyst	At 28 weeks' gestation
Breitowicz et al. [10]	1	14	5-months	GROWING frequency and intensity	Vomiting	–	US	–	Laparascopy	Both	Both 2	Bilateral salpingectomy	5.5 * 7.0 cm^2^ and 5.5 * 9.3 cm^2^	Cysts recognized as paramesonephric cysts	Both side torsion
	2	17	–	Progressing turn to suddenly pain	Vominting + couple of months abdominal pain after intercourse	–	US	–	Laparascopy	Left	4	Salpingectomy	4.0 * 5.0 cm^2^	Recognized as a paramesonephric cyst	Sexually active, using hormonal contraception
Grover [24]		11.5	28-h	Progressiively worsening	–	–	US	Torted ovarian cyst, hemorrhagic ovarian cyst, or appendix abscess	Laparascopy	Right	1	Salpingectomy	5 * 5 cm^2^	–	
Said et al. [25]		12	2-days	Sudden in onset	Vomiting + tachycardia	WBC 18.660/ml	US	Torsion	Laparoscopy	Right	3	Untwisted	9.76 * 9.1 cm^2^	Right mesothelial cysts	Long-standing history of being overweight, BMI42
Seshadri et al. [11]		12	2-day	–	Vomitng	WBC 18,000/ml	US	Appendicular mass	Laparoscopy	Right	–	Salpingectomy	7.7 * 6.5 cm^2^ and 8^*^4.6 cm^2^	Benign	
Rajaram et al. [26]		18	7-day	Aggravation the last 2 days	–	Normal	US	–	Laparoscopy	Right	2.5	Salpingectomy	4 × 4 cm^2^	Paratubal cyst lined by mesothelium	
Blitz et al. [3]		14	–	–	Vomiting	WBC 8800/mL	US + CT	–	Laparoscopy	Right	–	Salpingectomy	2 cm in diameter	–	Tube associated with noncommunicating rudimentary horn
Ryu et al. [17]		70	–	Acute	–	CRP 29.5 mg/L, CA 19–9 1146 IU/ml,	US + CT	Adnexal torsion	Laparoscopy	Both	Right: 1; left: 2	Radical resection	4 cm right, 6 cm left	Serous papillary adenocarcinoma; paratubal cyst of mesothelial origin	Malignant paratubal cancers and both torsion
Radoica Jokić et al. [16]		16	2-days	Acute	–	Normal	US	Torsion of the right adnexal	Laparoscopy	Right	2	Appendectomy and untwist tube	5 × 4 cm^2^	Chronic appendicitis and simple paratubal cyst	Received hormonal therapy for PCOS; with appendicitis
Gunal et al. [27]	1	12	3-days	Acute	–	WBC 12,300/ml	US	–	Laparotomy	Left	2	Untwisted	5 * 5 cm^2^	Simple cyst lined by single layer of tubal type epithelium with hemorrhages	
	3	13	4-h	Acute	–	Normal	US	Tubal torsion	Laparoscopy	Right	–	Untwisted	5.5 cm in diameter	MESONEFRIC paratubal cyst	
Yeamie et al. [14]		13	2 days	Bearable to intolerable	Vomiting + diarrhea	–	US + MRI	Tubal torsion according to MRI	–	Right	–	–	–	–	–
Ottino et al. [28]		11	1-day	Intermittent to worsened	–	Normal	US	–	Laparoscopic	Right	2	Salpingectomy	5.7 * 3.9 cm^2^	Benign paratubal cyst with a dilated fallopian tube	–
Takeda et al. [29]		30	Seven hours	Acute	Vomiting + uterine contractions occurred	WBC 13.100/ml	US + MRI	Isolated tubal torsion due to MRI	LESS surgery	Right	–	Salpingectomy	4 cm in diameter	Paratubal cyst	At 30 + 5 weeks' gestation
Ours		13	2 episode pain during 12 days	Acute in last 2 days	–	Normal	US	Tubal torsion	Laparoscopic	Right	3	Untwisted	5.5 cm in diameter	Serous cystadenoma	

Blood tests were reported in 16 patients, six patients showed leukocytosis, and one patient showed elevated CRP and CA199. Nineteen patients had the surgical approaches recorded, and these included 13 laparoscopic surgeries and six laparotomies. The most common procedure performed (13 cases) was salpingectomy, and one of the procedures was a radical operation performed due to malignant paraovarian cancer found by rapid pathological examination. Conservative surgical management was performed in six out of 19 cases, in which the salpinges appeared viable after detorsion. Histopathologic diagnosis was reported in 17 cases; only six cases had definitive diagnoses, including three cases of mesothelial origin, one case of mesonephric origin and two cases of paramesonephric origin.

## Discussion and conclusions

**Fig. 4:**
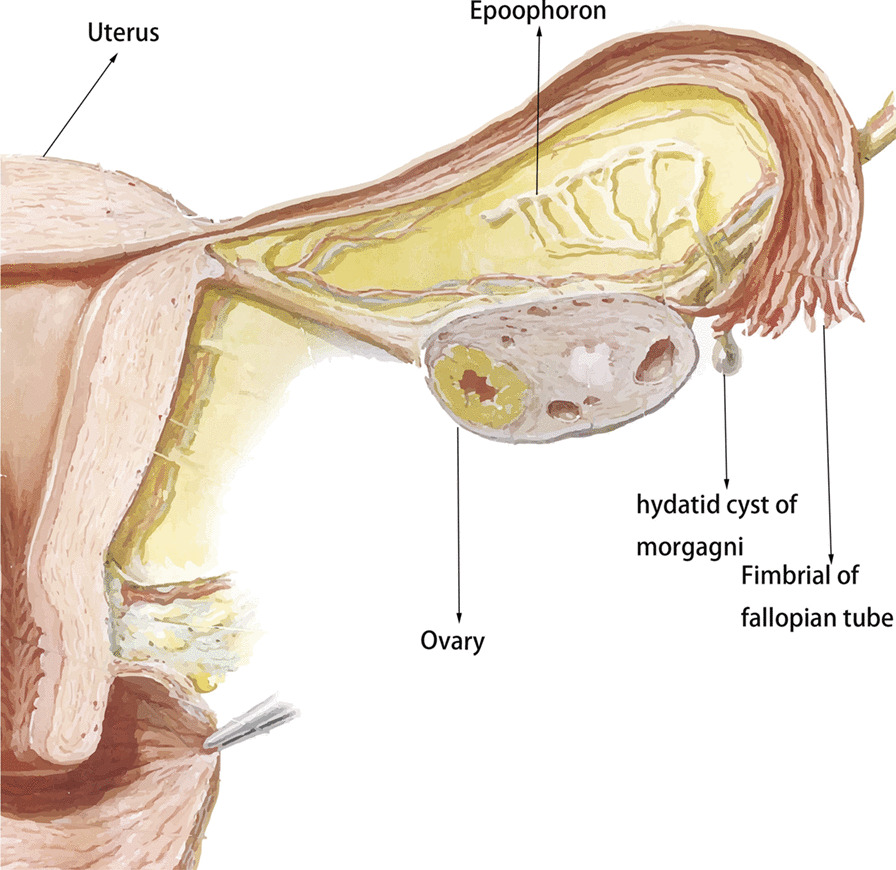
Anatomical details of the female internal genitalia

Paraovarian or paratubal cysts are present in approximately 10% of all adnexal masses [6]. They arise from epoophores, which are located in the broad ligament and consist of a longitudinal ductulus and 10–20 transverse ductuluses, and these ductuli are secretory (Fig. 4). Paraovarian cysts should be distinguished from Morgagni’s hydatid cysts, which are usually smaller in size and located in the fimbriated end of the fallopian tube. Paraovarian cysts are generally unilocular, contain clear fluid and are of paramesonephric, mesothelial, or mesonephric origin [7]. Histopathology may show secretory and ciliated cells (paramesonephric origin), low cuboidal epithelium and occasional clear cells (mesonephric origin), lined by flattened epithelium with occasional tubal differentiation and surrounding fibrous tissue (mesothelial origin). Because distention of the cavity often distorts the epithelium, absolute differentiation is difficult [8]. According to the results of this study, only 6/20 cases determined the origins of the cysts.

In general, parovarian cysts could be found at any age, and most of them were benign tumors. Paraovarian cysts are usually detected by ultrasonography or during surgery. Nevertheless, due to hormonal activity, especially in adolescence or during pregnancy, cystic dilatation often occurs [9]. In this study, we found that the average diameter of cysts when torsion occurred was approximately 5-cm (the minimum was 2-cm, and the maximum was 9-cm); the median age was 15 years; 13/20 of the patients were adolescents; 6/20 patients were adults; and 1/20 was postmenopausal. In addition, 5/20 cases had torsions that occurred during pregnancy (four cases) or puerperium (one case). Cystic distention could increase the mobility of adnexal tissues, which may induce a reversal, particularly when the cysts reach 5-cm [10]. The cysts can torse on their own or can predispose to isolated tubal torsion. The distinction between the two situations before surgery is challenging, and the main clinical manifestation is abdominal pain.

Patients presented with abdominal pain, which could be described as gradual or acute, persistent or intermittent, and mild or severe. We found that 17 cases had the mode of pain recorded, and all of them presented with emergency abdominal pain, such as acute pain, severe pain, and aggravated pain. Some of the cases had a history of previous mild pain that subsequently worsened. Associated symptoms were variable and the most frequent symptom was vomiting (9/20), which was considered a stress response to the intense abdominal pain. On physical examination, mild tenderness was present in all patients, and peritoneal signs developed in advanced cases (if the condition progressed) [11]. The median duration time of pain was 3 days. Mazouni et al. [12] reported that the risk of adnexal necrosis was increased significantly when the delay time for surgery was over 10 h. A previous study also found that patients with pain for more than 24 h were more likely to undergo salpingectomy, suggesting that longer periods of torsion can lead to more tissue necrosis [5]. Moreover, we observed that nine patients presented with vomiting, 8/9 patients had their procedure reported, and 7/8 patients underwent salpingectomy. This may demonstrate that the amount and severity of necrosis from the torsion is related not only to the duration of pain but also to the degree of pain, which probably represents the varying degrees of torsion. Until now, most importantly, timely diagnosis and surgical intervention have remained the most effective ways to avoid salpingectomy.

The majority of fallopian tube torsions occurred on the left (14/20). The most likely explanation was that the sigmoid colon on the left provides cushioning as an accessory to prevent torsion by limiting the torsional activity, and patients with right abdominal pain more often underwent surgery because of the suspicion of appendicitis [13]. Fifteen of 20 cases had the number of torsion rotations recorded, and the average number of torsion rotations was 2.38 rotations. There was no significant difference in torsion time between the salpingectomy group and preservation group (*P* = 0.651), demonstrating that necrosis was not associated with torsion times.

Ultrasonography was performed as the first diagnostic examination in all cases. The most specific characteristic of imaging of IFTT with paraovarian cysts was the presence of normal ovaries and a cystic mass, with or without a dilatated fallopian tube mass, which was due to tubal edema due to the torsion. The “break sign” was also another specific sign of IFTT, which showed narrowing at the extremity of the tube due to the torsion [14]. Color Doppler ultrasonography can detect tubal blood flow, which can also be detected in cases of incomplete obstruction, so the presence of flow cannot completely rule out torsion. However, a high impedance waveform with reversal of diastolic flow could be helpful, which implies torsion of the tube [15]. Compared with ultrasound, MRI could more clearly show findings such as dilated tubes, beak signs, and twisted pedicles. In this study, two patients were diagnosed by MRI as having IFTT with paratubal cysts before surgery. Radoica Jokić et al. [16] reported that MRI and ultrasound could provide credible information without the risks of radiation, which is especially helpful for pregnant patients. If a papillary projection on the cyst wall is present in sonographic or MRI images, malignant tumors and cystadenomas should be considered despite the low incidence of paraovarian cystic cancers [17].

In this study, most patients (13/19) underwent salpingectomy, and only six patients underwent more conservative surgical management, including detorsion of the tube and removal of paraovarian cysts. Surgical decisions depended on the observations of surgeons during the operation and whether the tubes developed normal circulation without necrosis after detorsion. Bertozzi et al. [18] reported that conservative management for IFTT could also be considered in cases of necrotic tubes because morbidity would not increase, but it could create the possibility of leaving a nonfunctional tube. Considering that the majority of patients were adolescents, tubal conservation should be favored as much as possible, because of the concerns for fertility in the future.

## Data Availability

All data generated or analyzed during this study are included in this published article.

## Abbreviations

IFTT: Isolated fallopian tube torsion
ICU: Intensive care unit
